# The application of nonsense-mediated mRNA decay inhibition to the identification of breast cancer susceptibility genes

**DOI:** 10.1186/1471-2407-12-246

**Published:** 2012-06-15

**Authors:** Julie K Johnson, Nic Waddell, Georgia Chenevix-Trench

**Affiliations:** 1 Queensland Institute of Medical Research, Brisbane, Australia; 2 School of Medicine, University of Queensland, Brisbane, Australia; 3 Queensland Centre for Medical Genomics, Institute for Molecular Bioscience, The University of Queensland, Brisbane, Australia; 4 The Kathleen Cuningham Foundation for Research into Familial Breast Cancer (kConFab), Peter MacCallum Cancer Centre, Melbourne, Australia; 5 Queensland Institute of Medical Research, Royal Brisbane Hospital, Locked bag 2000, Herston, Brisbane, QLD, 4029, Australia

## Abstract

**Background:**

Identification of novel, highly penetrant, breast cancer susceptibility genes will require the application of additional strategies beyond that of traditional linkage and candidate gene approaches. Approximately one-third of inherited genetic diseases, including breast cancer susceptibility, are caused by frameshift or nonsense mutations that truncate the protein product [1]. Transcripts harbouring premature termination codons are selectively and rapidly degraded by the nonsense-mediated mRNA decay (NMD) pathway. Blocking the NMD pathway in any given cell will stabilise these mutant transcripts, which can then be detected using gene expression microarrays. This technique, known as gene identification by nonsense-mediated mRNA decay inhibition (GINI), has proved successful in identifying sporadic nonsense mutations involved in many different cancer types. However, the approach has not yet been applied to identify germline mutations involved in breast cancer. We therefore attempted to use GINI on lymphoblastoid cell lines (LCLs) from multiple-case, non- *BRCA1*/*2* breast cancer families in order to identify additional high-risk breast cancer susceptibility genes.

**Methods:**

We applied GINI to a total of 24 LCLs, established from breast-cancer affected and unaffected women from three multiple-case non-*BRCA1*/*2* breast cancer families. We then used Illumina gene expression microarrays to identify transcripts stabilised by the NMD inhibition.

**Results:**

The expression profiling identified a total of eight candidate genes from these three families. One gene, *PPARGC1A*, was a candidate in two separate families. We performed semi-quantitative real-time reverse transcriptase PCR of all candidate genes but only *PPARGC1A* showed successful validation by being stabilised in individuals with breast cancer but not in many unaffected members of the same family. Sanger sequencing of all coding and splice site regions of *PPARGC1A* did not reveal any protein truncating mutations. Haplotype analysis using short tandem repeat microsatellite markers did not indicate the presence of a haplotype around *PPARGC1A* which segregated with disease in the family.

**Conclusions:**

The application of the GINI method to LCLs to identify transcripts harbouring germline truncating mutations is challenging due to a number of factors related to cell type, microarray sensitivity and variations in NMD efficiency.

## Background

Germline mutations in either one of the two major breast cancer tumour suppressor genes, *BRCA1* or *BRCA2*, confer a 60-85% lifetime risk of breast cancer but explain only around 20% of the breast cancer cases that have a family history of the disease [2-8]. However, almost 70% of breast cancer families with four or more cases of early onset breast cancer under the age of 60 show no convincing evidence of linkage to *BRCA1* or *BRCA2*[9]. Nonetheless, for women without mutations in *BRCA1* or *BRCA2*, family history remains a strong predictive risk factor for breast cancer [10-12]. This unexplained familial aggregation of the disease suggests the presence of additional high-risk, breast cancer susceptibility genes, particularly in non-*BRCA1/2* families with many cases of early onset breast cancer [13].

The largest genome-wide breast cancer linkage study since the identification of *BRCA2*, conducted on 149 non-*BRCA1*/*2* breast cancer families by the Breast Cancer Linkage Consortium, failed to find any significant linkage signals [14]. However, the selection criteria of the families, differences in the population background, or clinical and genetic heterogeneity might determine the power to detect a linkage signal. Rosa- Rosa et al performed linkage analysis in 41 breast-cancer Spanish families and found a significant linkage signal (HLOD score 3.55) at 21q22. They also found a HLOD of 3.13 on the 3q25 region [15] in a subset of 13 families with bilateral breast cancer. Collectively, the published linkage studies [15-17] do not provide conclusive evidence that high risk *BRCA*-like genes exist, but certainly indicate that if they do, their mutations would only account for a small fraction of the non-*BRCA1*/*2* families. Candidate gene approaches for mutation screening rely on *a priori* information about biological gene function and are thus limited by how much is known about the biology of the disease. Identification of rare mutations in highly penetrant breast cancer susceptibility genes will therefore require the application of more novel strategies beyond that of traditional linkage and candidate gene approaches. A strategy for disease gene identification by NMD inhibition (GINI) utilising gene expression profiling has been developed to identify dysregulated transcripts which may carry protein truncating mutations, thereby allowing this approach to be used to prioritise genes for mutation analysis [18].

The Breast Cancer Information Core database (BIC; http://research.nhgri.nih.gov/bic/; version modified September 2010) states that almost 50% of all reported *BRCA1* mutations and about 30% of reported *BRCA2* mutations are either frameshift or nonsense mutations, thus it is reasonable to expect that protein truncating mutations will be common in any other highly penetrant breast cancer susceptibility genes. The NMD pathway selectively and rapidly degrades mutant messenger RNAs harbouring premature termination codons (PTCs) [19-21]. Therefore, blocking the NMD pathway will stabilise these mutant transcripts, which can then lead to the identification of potential tumour suppressor genes that contain nonsense mutations [18,22]. Such genome-wide screens for transcripts harbouring truncating mutations has proved successful in identifying sporadic nonsense mutations involved in colon cancer [18,19,23,24], prostate cancer [25,26], melanoma [27], mantle cell lymphoma [28] and clear cell renal cell carcinoma [29]. However, the approach has not yet been applied to identify germline mutations involved in breast cancer. In an attempt to identify additional high-risk genes involved in familial breast cancer, we have applied this disease-gene identification technique to affected and unaffected members of multiple- case non-*BRCA1*/*2* breast cancer families.

## Methods

### Selection of non-BRCA1/2 breast cancer families

The Kathleen Cuningham Foundation Consortium for Research into Familial Breast Cancer (kConFab; http://www.kconfab.org) provides a comprehensive resource upon which researchers can draw data and biospecimens for peer-reviewed, ethically- approved funded research projects on familial aspects of breast cancer. We selected three families (Family A, B and C) for use in GINI (Table 1), in which no *BRCA1* or *BRCA2* mutations had been found, and which had Manchester scores >19 [30,31] suggesting a high probability of having a mutation in a breast cancer susceptibility gene. For each family, there were at least three LCLs from women affected with breast cancer at ages 34–56, and at least three from women who were unaffected at ages 26–72. Ethical approvals were obtained from the Human Research Ethics Committees of the Queensland Institute of Medical Research and the Peter MacCallum Cancer Centre. Written informed consent was obtained from each participant.

**Table 1:** **Multiple-case non-*BRCA1*/*2*breast cancer families chosen for GINI analysis**

**Family**	**Manchester Score*****BRCA1BRCA2***		**Number of LCLs from individuals affected with breast cancer**	**Ages of affected individuals at the time of their diagnosis**	**Number of LCLs from individuals unaffected by breast cancer**	**Ages of individuals unaffected by breast cancer**	**Total number of samples hybridised to gene expression array**
A	20	19	5	26, 34, 43, 47, 64	3	32, 38, 63	48
B	24	21	3	39, 39, 41	4	26, 28, 57, 58	42
C	19	19	3	36, 44, 56	6	41, 45, 62, 68, 70, 72	54

### Cell lines

We used the colon cancer cell line, HT29, which contains a truncating mutation (c.931C > T p.Q311X) in one allele of the *SMAD4* gene and a deletion of the other allele, as a positive control, as well as two positive control LCLs (*BRCA1* c.2681_2682delAA and *BRCA2* c.539_541insAT). These truncating mutations occur >55 nucleotides upstream from the final exon of each gene and are thus expected to undergo nonsense-mediated mRNA decay. To identify putative novel, breast cancer susceptibility genes by GINI, we used 24 LCLs established from patients affected with breast cancer and unaffected individuals from three non-*BRCA1*/*2* breast cancer families.

### Gene inhibition of nonsense mediated decay (GINI)

Twenty-four hours prior to caffeine treatment, we seeded HT29 cells into two 75cm^2^ cell culture flasks, each containing 1x10^6^ cells in 10mls of tissue culture medium (RPMI-1640 + 10% FBS). We then added fresh medium containing 10mM caffeine (Sigma-Aldrich, St. Louis, MO, USA) to one flask and fresh medium without caffeine to the control flask. After four hours of incubation at 37°C, we removed the medium from both flasks and washed the cells twice with phosphate-buffered saline (PBS). We then added normal medium to the untreated control flask and treated the other flask for a further four hours with 10mM caffeine medium. We then removed the media from both flasks and washed the cells twice with PBS before storing the cells at −80°C until RNA extraction. We repeated this process ten times to ensure repeatability of results. We then performed semi-quantitative real-time reverse transcriptase (RTPCR) of *SMAD4* to confirm stabilisation of the target gene after caffeine treatment, and cRNA from four randomly selected replicates were then hybridised to the Illumina HumanHT-12 v3 gene expression arrays.

In order to determine the optimal caffeine concentration for treating LCLs, we treated 3.5x10^6^ LCLs with 2.5mM, 5mM, 7.5mM, 10mM or 15mM caffeine (Sigma-Aldrich) for two lots of four-hour incubations at 37°C in the same GINI process described above for HT29 cells. We determined the optimal concentration by semi-quantitative real-time RT-PCR of *BRCA1* and *BRCA2* (Additional File 1) and then performed three technical replicates of GINI on the positive control LCLs on different days, and hybridised the cRNA to Illumina HumanHT-12 v3 gene expression arrays.

### GINI on LCLs from non-BRCA1/2 breast cancer families

Having optimised the GINI method on positive control LCLs with *BRCA1* or *BRCA2* mutations, we applied it to LCLs from 11 affected and 13 unaffected individuals from three non-*BRCA1*/*2* breast cancer families, in parallel with positive control HT29 cells. We performed three technical replicates for each sample on different days and then hybridised cRNA from caffeine-treated and untreated control samples to Illumina HumanHT-12 v3 gene expression arrays.

### Semi-quantitative real-time reverse transcriptase PCR (RT-PCR) and expression array profiling

We extracted total RNA from frozen cell pellets using the RNeasy RNA Extraction Kit (Qiagen, Hilden, Germany) as per the manufacturer’s instructions. We used semi- quantitative real-time reverse transcriptase PCR (RT-PCR) to validate the expression of the target genes in the positive controls and of the candidate genes found by the GINI method. Primer sequences are provided in Additional File 2. We synthesised cDNA from 1μg of total RNA in a reaction volume of 20μl using oligodT and Superscript III (Invitrogen) according to manufacturer’s instructions. The cDNA reactions were then diluted to 200μl (1:10) in RNase/DNase-free water. We performed semi-quantitative real-time RT-PCR amplifications in quadruplet on the LightCycler480 (Roche) machine using 3.75μl SYBR Green PCR Master Mix (Invitrogen) and 0.33μM forward and reverse primers in a total reaction volume of 7.5μl, using the glyceraldehydes-3-phosphate dehydrogenase (*GAPDH*) gene as a reference. We normalised the comparative threshold-cycle (Ct) of signal intensities of amplified product to that of *GAPDH* calibrating to the matched untreated control of each sample using the LightCycler480 software program (Roche Diagnostics Corp., Indianapolis, IN, USA).

For the expression profiling, we prepared biotinylated cRNA from 450ng of total RNA using the IlluminaTotalPrep RNA Amplification Kit (Ambion, Austin, TX, USA) and hybridised 750ng cRNA per sample to HumanHT-12 v3 Expression BeadChips (Illumina Inc., San Diego, CA, USA) as per the Whole-Genome Gene Expression Direct Hybridisation Assay protocol. We collated expression data using BeadStudio Version 1.5.1.3 (Illumina Inc.) and then quantile normalised the raw data using the R-Bioconductor LUMI package [32]. Microarray data were submitted to Gene Expression Omnibus (GEO) [33] and are accessible through GEO (http://www.ncbi.nlm.nih.gov/geo/query/acc.cgi?acc=GSE37210). To identify differentially expressed genes, between each caffeine treated sample to its own untreated control over three technical replicates, we implemented a linear model and empirical Bayes method using the R-Bioconductor LIMMA package [34]. We considered genes with Benjamini and Hochberg adjusted P-values less than 5% that also have a greater than 50% chance of being differentially expressed (positive B- statistic) as having statistically significant changes in their expression profiles. We imported differentially expressed genes into GeneSpring v10.0 (Agilent Technologies) for visualisation and further analysis.

We defined genes that were differentially expressed between all untreated and all caffeine treated samples as “global caffeine response” genes. These genes are unlikely to contain truncating mutations in breast cancer susceptibility genes but are more likely to be expressed in response to caffeine or be naturally occurring NMD targets.

For each family, we selected candidate genes for semi-quantitative real-time RT-PCR validation and mutations screening that were upregulated after caffeine treatment in the individuals affected with breast cancer but were not differentially expressed after caffeine treatment in their unaffected family members, and therefore not part of the “global caffeine response” gene list.

### Sequence analysis

We sequenced the exonic and flanking splice sites of the one candidate gene we identified by GINI in the relevant family. We amplified 500ng DNA by polymerase chain reaction (PCR) with 1X PCR Buffer, 2.5mM MgCl_2_, 0.2mM dNTP, 0.25μM forward and reverse primer, 0.4μl AmpliTaq Gold in a total reaction volume of 50μl. We then purified the PCR products using the QIAquick PCR Purification Kit Spin Protocol (Qiagen) prior to sequencing according to manufacturer’s instructions using BigDye Terminator v3.1 and an ABI 3100 Genetic Analyser (PE Applied Biosystems, Foster City, CA, USA).

### Haplotyping around the PPARGC1A region

We used a total of six short tandem repeat microsatellite markers from a 2Mb region around *PPARGC1A* to haplotype around the *PPARGC1A* gene in members of Family B. We selected four markers (D4S3017, D4S2953, D4S425, D4S23013) (deCODE Genetics; http://www.decode.com) and designed two (19xTA and 20xTG) more around short tandem repeats within the DNA sequence of *PPARGC1A* and labelled either the forward or reverse primer with a 6-FAM label. We amplified 500ng DNA by polymerase chain reaction (PCR) with 1X PCR Buffer, 2.5mM MgCl_2_, 0.2mM dNTP, 0.25μM forward and reverse primer, 0.4μl AmpliTaq Gold in a 50μl final volume. Amplified PCR products were diluted 1:800 with Hi-Di formamide/500 LIZ size standard mix (formamide to size standard ratio of 67:1) (Applied Biosystems) and the mixture was then denatured for 5 min at 95°C. The samples were separated in an ABI PRISM3700 DNA Sequencer and subjected to the fragment analysis protocol. Allele scoring was performed using the GeneMapper 4.0 software (Applied Biosystems).

## Results

### Optimisation of GINI

**Figure 1:**
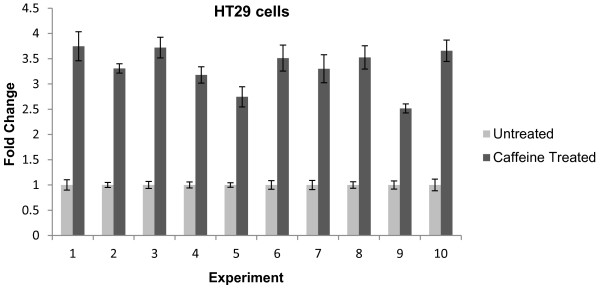
**Stabilisation of*SMAD4*mRNA in HT29 cells after caffeine (10mM) treatment measured by qRT-PCR.** Each caffeine treated sample has been normalised to the housekeeping gene, *GAPDH*, and calibrated to its untreated equivalent. Error bars represent standard error of the mean of four technical replicates of each PCR reaction.

We used HT29 cells to test the robustness of the GINI technique with caffeine treatment. Results from semi-quantitative real-time RT-PCR validation of ten replicate experiments identified a consistent 2.5- to 3.7-fold upregulation of *SMAD4* mRNA after 10mM caffeine treatment (Figure 1). Microarray results of three replicates identified a total of 553 probes corresponding to 495 genes that were significantly differentially expressed ≥2-fold between untreated and caffeine-treated HT29 cells. When sorted by adjusted P-value, *SMAD4* ranked as the 48^th^ most differentially expressed gene and the 28^th^ most significantly upregulated gene (fold change = 2.78; adj P-value = 7.83-E07; B-statistic = 12.94).

**Figure 2:**
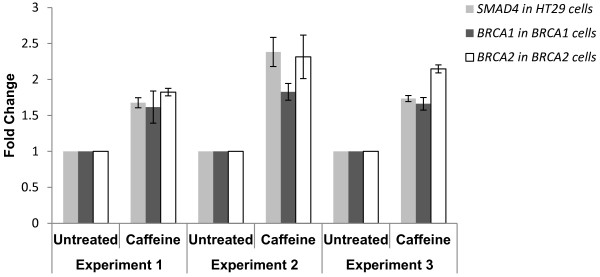
**Stabilisation of target genes in HT29, BRCA1 and BRCA2 positive control cell lines after GINI with caffeine (7.5mM) treatment.** Three experimental replicates of the GINI method were performed on HT29 cells, BRCA1 c.2681_2682delAA and BRCA2 c.539_541insAcontrol LCLs. qRT-PCR results indicate that each experimental replicate shows at least a 1.5-fold stabilisation of all three target genes after treatment with 7.5mM caffeine. Each sample has been normalised to the housekeeping gene, *GAPDH*, and calibrated to its untreated equivalent. Error bars represent standard error of the mean of four technical replicates of each PCR reaction.

In parallel with positive control HT29 cells, we treated LCLs with known *BRCA1* or *BRCA2* mutations with a caffeine concentration gradient and used semi-quantitative real-time RT-PCR to find the concentration that best stabilises their respective target genes (Additional File 1). The highest level of target gene stabilisation in the LCLs was 2.4-fold at 7.5mM caffeine. We repeated the GINI experiments a total of three times for the *BRCA1* and *BRCA2* cell lines, and each replicate of every cell line showed at least a 1.5-fold stabilisation of their respective target genes after treatment with 7.5mM caffeine (Figure 2).

### Identifying candidate breast cancer susceptibility genes by GINI in nonBRCA1/2 breast cancer families

We applied GINI, with a caffeine concentration of 7.5mM, to 24 LCLs from three non-*BRCA1/2* families. We identified a set of 6,520 global caffeine response” genes which were differentially expressed (adjusted P-value < 0.0006 and B-statistic > 0) between untreated and caffeine treated samples from all individuals combined from the three families. Of these “global caffeine response” genes, 1,364 and 292 genes changed more than 1.5- and 2-fold after caffeine treatment, respectively.

**Figure 3:**
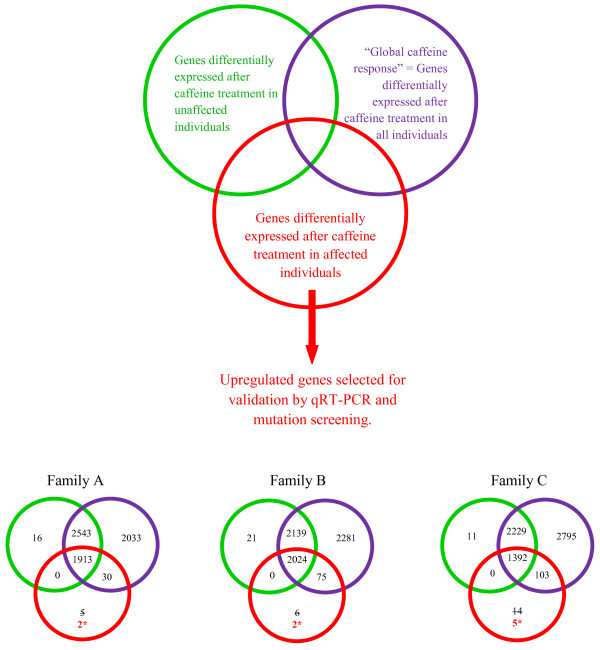
**Identification of candidate breast cancer susceptibility genes for mutation screening in multiple-case non-*BRCA1*/*2*breast cancer families.** For each family, candidate genes included those that were upregulated after caffeine treatment in individuals affected with breast cancer but not in their unaffected family members and also not part of the “global caffeine response” gene list.

We identified candidate protein truncated transcripts within each family as those with statistically significant increased expression after caffeine treatment in affected individuals of that family, but not in unaffected individuals of the other two families, and which were not part of the “global caffeine response” (Figure 3). We identified a total of two, two and five candidate genes for Family A, B and C, respectively (Table 2). Interestingly, only one gene, peroxisome proliferator-activated receptor-γ coactivator-1 α *(PPARGC1A)*, was identified as a candidate in more than one family.

**Table 2:** Candidate genes identified by GINI analysis

**Family**	**Gene Name**	**Fold Change**	**Adjusted P-value**	**B-statistic**
A	*WNT5A*	1.14	0.005560	0.04
	*RAB3B*	1.17	0.004254	0.33
B	***PPARGC1A***	1.19	**0.001226**	4.23
	*CD14*	1.19	0.001875	1.19
C	***PPARGC1A***	1.41	**3.00E-11**	25.02
	*METRNL*	1.44	0.001146	2.51
	*BMP6*	1.43	3.61E-05	6.67
	*PRDM1*	1.25	0.000314	4.04
	*GRSF1*	1.69	0.007637	0.23

### Semi-quantitative real-time RT-PCR and sequencing of candidate breast cancer susceptibility genes

**Figure 4:**
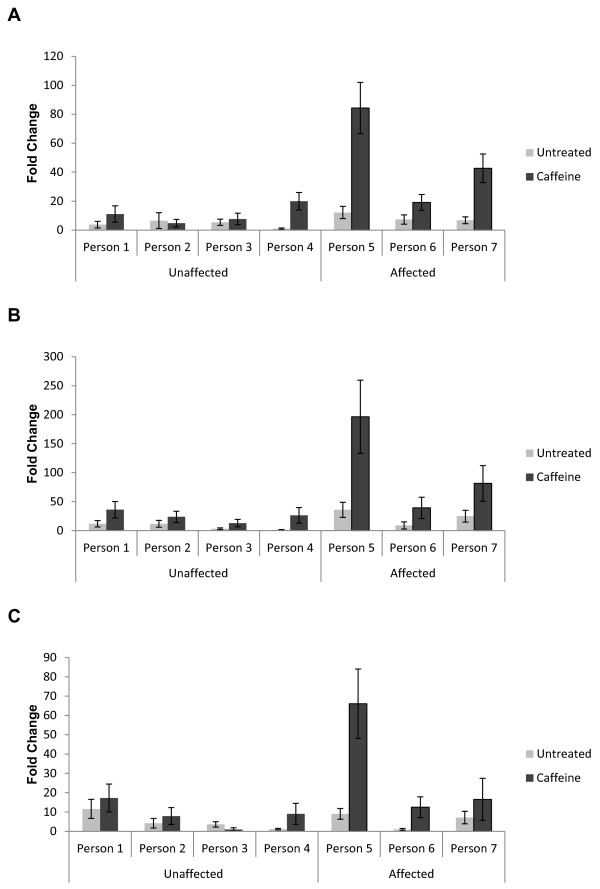
**Stabilisation of*PPARGC1A*mRNA in the lymphoblastoid cell lines (LCLs) of individuals from Family B after caffeine (7.5mM) treatment measured by semi-quantitative real-time RT-PCR.***PPARGC1A* mRNA is stabilised in family members affected by breast cancer and not in unaffected family members, with the exception of Person 4. Each sample has been normalised to the housekeeping gene, *GAPDH*, and calibrated to the lowest expressing untreated sample. Standard error bars represent standard error from the mean of four technical replicates for each PCR reaction.

**Figure 5:**
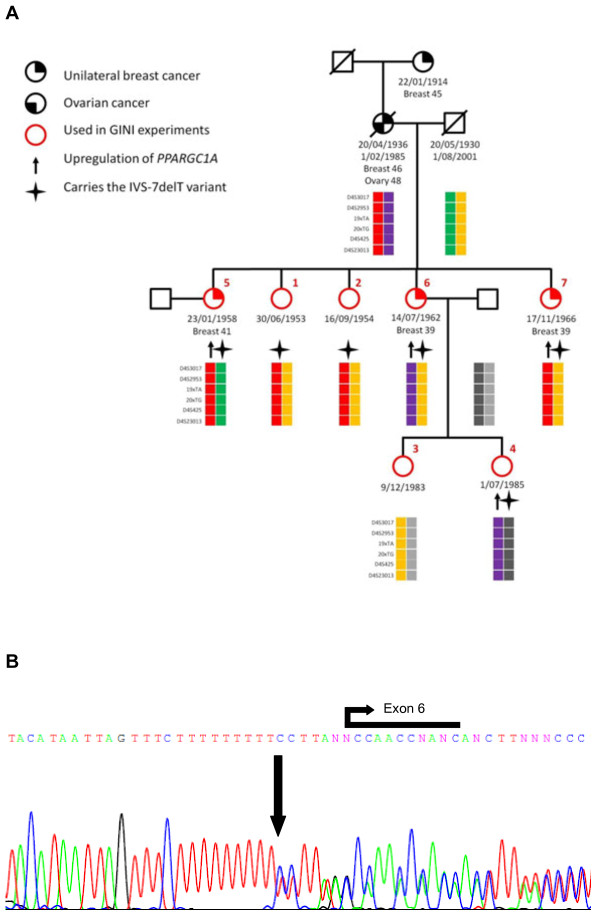
**Pedigree of Family B (A) and sequencing chromatogram of*PPARGC1A*IVS-7delT variant (B).** GINI experiments were performed on the LCLs derived from individuals marked with red circles. The red coloured numbers correspond to the person number in Figure 4. Upward arrows indicate individuals that showed stabilisation of the *PPARGC1A* transcript. The IVS-7delT variant (B) is located seven base pairs upstream from the start of exon 6 and was found in both affected and unaffected members of Family B as indicated by stars in the pedigree.

We tried to validate all nine candidate genes in with semi-quantitative real-time RT-PCR but only *PPARGC1A* showed consistent stabilisation of mRNA transcript across replicates of Family B in individuals affected by breast cancer and not in their healthy family members (Figure 4 and Additional File 3,9, Additional File 4, Additional File 5, Additional File 6, Additional File 7, Additional File 8). Primer sequences are provided in Supplementary Table 1. The youngest individual in Family B (individual 4: aged 26) showed statistically significant stabilisation of the *PPARGC1A* transcript in two out of three experimental replicates, suggesting that this individual may be a carrier for a family-specific breast cancer mutation but has not yet developed disease. However, sequencing of all coding regions in all affected and unaffected family members and flanking splice sites did not reveal any protein truncating mutations in *PPARGC1A*. We identified three coding (rs2970847, rs3755863 and rs8192678) and one non-coding (rs2946385) single nucleotide polymorphisms (SNPs). The only variant identified that was not listed in dbSNP as a common polymorphism was IVS7delT (Figure 5). However, this variant was found in all members sequenced including affected and unaffected individuals. Furthermore, Human Splicing Finder [35] suggested that this intronic variant has no effect on splicing. Haplotype analysis using short tandem repeat microsatellite markers spanning 2Mb around *PPARGC1A* showed no evidence of a haplotype that might carry a protein truncating mutations segregating with disease within the family (Figure 5).

## Discussion

In an attempt to identify germline mutations in additional high risk breast cancer susceptibility genes, we have optimised and applied the GINI method on lymphoblastoid cell lines derived from the blood of women from multiple-case non- *BRCA1*/*2* breast cancer families. By using positive control cell lines with known truncating mutations, we have determined the optimal concentration of caffeine that results in significant stabilisation of target genes, which is suggestive of successful inhibition of the nonsense-mediated mRNA decay pathway. Microarray analysis of the transcripts stabilised after NMD inhibition by caffeine treatment in women affected with breast cancer compared to their unaffected relatives identified a total of eight different genes across three families. One gene, peroxisome proliferator- activated receptor-γ coactivator-1 α (*PPARGC1A*), was a candidate gene in two families and was the only breast cancer susceptibility candidate gene that we could demonstrate by semi-quantitative real-time RT-PCR as being consistently upregulated after GINI in affected members of the family, but not in most unaffected relatives.

*PPARGC1A* is a master transcriptional *regulator of mitochondrial oxidative phosphorylation and cellular energy metabolism.* The gene is expressed in a broad range of tissues with higher levels of expression detected in tissues with high oxidative capacity, such as heart, skeletal muscle, brown adipocyte, kidney and brain [36-38]. Upregulation of *PPARGC1A* in response to oxidative stress can suppress the production of reactive oxygen species [39]. PPARGC1A also plays an important role as an estrogen receptor coactivator in the estrogen receptor (ER) pathway by binding and enhancing transactivation of estrogen receptor alpha (ERα) in a ligand-dependent manner [40,41]. Persistent estrogen mediated mitogen signalling of ERα has been known to stimulate the growth of a large proportion of breast cancers [42-45]. In fact, over half of all breast cancers overexpress ERα [46]. An association study of ~800 *BRCA1*/*2* mutation-negative familial breast cancer cases and over 1,000 controls from Germany found some evidence that the *PPARGC1A* Thr612Met polymorphism might be a risk factor for familial breast cancer (OR = 1.35, 95% CI 1.00-1.81, *P* = 0.049), high-risk familial breast cancer (OR = 1.51, 95% CI 1.08-2.12, *P* = 0.017) and bilateral familial breast cancer (OR = 2.30, 95% CI 1.24-4.28, *P* = 0.009) [47]. However, haplotype analysis did not identify any additional association with familial breast cancer [47].

Although, we did not identify any truncating mutations in the coding or splice site regions of *PPARGC1A*, we did find an IVS-7delT variant in both affected and unaffected individuals of two families. However, haplotyping analysis around *PPARGC1A* did not identify a haplotype that segregated with disease in either family, which may contain a cryptic, deeply intronic, mutation that causes protein truncation.

Caffeine can impact on the alternative splicing of a subset of cancer-associated genes [48]. For example, caffeine can result in alternatively spliced isoforms of chaperonin- containing TCP1 subunit 3 (*CCT3*), asparagine synthetise (*ASNS*), COMM-domain containing 5 (*COMMD5*), ATP binding cassette subfamily F member 2 (*ABCF2), SLC39A1/ZIRTL*, and yippee-like 5 gene (*YPEL5*) being expressed. Exposure of HeLa cells to caffeine can also result in differential expression of 40 cancer-associated gene (for example, *KLF6SC35CCT3ASNSCOMMD5ABCF2YPEL5*) isoforms [48], although it is worth noting that different patterns of gene expression result from differing concentrations of caffeine [49]. Nevertheless, even though increased stability of mutant RNA is suggested to be more likely [18], GINI does not distinguish between the increased stability of the mutant transcript and the selective depletion of the normal transcript [50]. Therefore, if NMD inhibition by caffeine treatment did result in the stabilisation of one transcript and the reduction of another isoform, then unless the probes present on the microarray can distinguish between these isoforms, the net change in gene expression may not have been detected on the array platform. Furthermore, nonsense codons can reduce the abundance of nuclear mRNA without affecting the abundance of pre-mRNA or the half-life of cytoplasmic mRNA [51] and this might further reduce the sensitivity of GINI.

In order to acquire a more selective list of nonsense transcripts for a particular cell line, it may be necessary to combine the results of multiple different methods of NMD inhibition: 1) siRNA against *UPF1*[52,53], 2) caffeine treatment, and 3) emetine treatment, which inhibits the progression of the ribosome along the mRNA [29,54]. A major problem with using the GINI approach for identifying pathogenic mutations in yet-unidentified high-risk breast cancer genes in the germline DNA of individuals affected with breast cancer is that the mutation is expected to be present in a heterozygous state (at least, in an autosomal dominant disorder). The stabilisation of only one allele reaches the sensitivity limits of gene expression microarrays. Therefore, genes that may have been mutated but are expressed at a moderate to low level may have been excluded from detection. Tumour suppressor genes are usually inactivated during the process of tumorigenesis by a two-step process involving an inactivating mutation in the target gene accompanied by loss of the wildtype allele. However, in LCLs established from peripheral blood mononuclear leukocytes, the normal wildtype allele could mask the effects of GINI on the mutated allele thus reducing the efficiency of GINI [55]. Furthermore, LCLs may not provide an accurate representation of genes that are active in breast tissue, and if the putative breast cancer susceptibility gene is not expressed in LCLs then GINI will not work to identify susceptibility genes.

There is also evidence to suggest that NMD efficiency varies between different people with the same mutation [56,57], between different tissue types within an organism [58,59], and even between different strains of the same cell type [60]. It is possible that variable efficiencies of NMD can influence the clinical outcome of hereditary and acquired genetic disease and thus act as a genetic modifier of human genetic diseases.

Inhibition of the nonsense-mediated mRNA decay pathway followed by microarray analysis has been successfully applied to cancer cell lines to identify protein truncating mutations that may underlie sporadic forms of cancer [19,23,25-29,54]. The GINI method has recently been applied to the LCLs from six prostate cancer patients and their healthy brothers in order to identify susceptibility genes in hereditary prostate cancer [55]. However, despite sequencing 17 candidate genes, no truncating mutations were found. The GINI method has also failed to identify putative tumour suppressor genes in gastric cancer cell lines with siRNA against UPF1 [61]. It is commonly reported that the GINI strategy leads to a high number of false positives [19,21,23,26-28,55,61,62]. The novelty of our approach is the ability to identify transcripts stabilised by NMD inhibition in multiple breast cancer patients within a family and compare this gene list to the transcripts that are stabilised in multiple unaffected members of the same family in an attempt to reduce the number of false positive hits. Despite this analysis identifying few candidate genes per family, we did not identify any detectable nonsense mutations. Therefore, our GINI analysis also results in a high number of false positives. However, it is also possible that the mechanism underlying susceptibility to breast cancer in non-*BRCA1*/*2* families may not be due to truncating mutations in susceptibility genes.

## Conclusion

In summary, we applied the gene identification by nonsense-mediated mRNA decay inhibition (GINI) strategy to lymphoblastoid cell lines established from the blood of affected and unaffected members of three multiple-case non-*BRCA1*/*2* breast cancer families but we did not identify any nonsense mutations that may underlie the breast cancer risk in any of the three families investigated. The application of the GINI method to identify germline mutations is challenging due to limitations including microarray sensitivity in detecting small fold changes, and because of individual variations in nonsense-mediated mRNA decay efficiency [24,50,56,59,60,63].

With the plummeting costs of next generation sequencing technologies, sequencing of whole exomes and genomes is becoming a much more attractive method to identify rare, yet high risk, pathogenic mutations underlying human genetic disease.

## Abbreviations

NMD, nonsense-mediated mRNA decay; GINI, gene identification by nonsense-mediated mRNA decay inhibition; LCL, lymphoblastoid cell line; PTC, premature termination codon; LOH, loss of heterozygosity; RNAi, RNA interference; siRNA, small interfering RNA; PCR, polymerase chain reaction; RT-PCR, reverse transcriptase PCR; FBS, fetal bovine serum; EBV, Epstein-Barr virus; PBS, phosphate-buffered saline; kConFab, the Kathleen Cuningham Foundation Consortium for Research into Familial Breast Cancer; Ct, cycle threshold.

## Competing interests

The authors declare that they have no competing interests.

## Authors' contributions

JKJ carried out the experiments and drafted the manuscript. NW participated in the design of the study and advised on data analysis. kConFab provided all the sample material. GCT conceived of the study. All authors read and approved the final manuscript.

## Pre-publication history

The pre-publication history for this paper can be accessed here:

http://www.biomedcentral.com/1471-2407/12/246/prepub

## Supplementary Material

Additional file 1 **Optimisation of caffeine concentration for lymphoblastoid cell lines (LCLs).** Level of mRNA stabilisation for two biological replicates of *SMAD4* in HT29 (A), *BRCA1* in BRCA1 c.2681_2682delAA LCL (B), *BRCA2* in BRCA2 c.6275_6276delTT LCL (C) and *BRCA2* in BRCA2 c.539_541insAT LCL (D) after treatment with different concentrations of caffeine (untreated - 15mM). Error bars represent standard error of the mean. Click here for file

Additional file 2 Primer sequences used for semi-quantitative real-time reverse transcriptase PCR of candidate genes identified with the GINI technique. Click here for file

Additional file 3 **Stabilisation of*WNT5A*mRNA in the lymphoblastoid cell lines (LCLs) of individuals from Family A after caffeine (7.5mM) treatment measured by semi-quantitative real-time RT-PCR.** Each sample has been normalised to the housekeeping gene, *GAPDH*, and calibrated to the lowest expressing untreated sample to show variation in transcript expression across individuals. Standard error bars represent standard error from the mean of four technical replicates for each PCR reaction. Click here for file

Additional file 9 **Stabilisation of*GRSF1*mRNA in the lymphoblastoid cell lines (LCLs) of individuals from Family C after caffeine (7.5mM) treatment measured by semi-quantitative real-time RT-PCR.** Each sample has been normalised to the housekeeping gene, *GAPDH*, and calibrated to the lowest expressing untreated sample to show variation in transcript expression across individuals. Standard error bars represent standard error from the mean of four technical replicates for each PCR reaction. Click here for file

Additional file 4 **Stabilisation of*RAB3B*mRNA in the lymphoblastoid cell lines (LCLs) of individuals from Family A after caffeine (7.5mM) treatment measured by semi-quantitative real-time RT-PCR.** Each sample has been normalised to the housekeeping gene, *GAPDH*, and calibrated to the lowest expressing untreated sample to show variation in transcript expression across individuals. Standard error bars represent standard error from the mean of four technical replicates for each PCR reaction. Click here for file

Additional file 5 **Stabilisation of*CD14*mRNA in the lymphoblastoid cell lines (LCLs) of individuals from Family B after caffeine (7.5mM) treatment measured by semi-quantitative real-time RT-PCR.** Each sample has been normalised to the housekeeping gene, *GAPDH*, and calibrated to the lowest expressing untreated sample to show variation in transcript expression across individuals. Standard error bars represent standard error from the mean of four technical replicates for each PCR reaction. Click here for file

Additional file 6 **Stabilisation of*PPARGC1A*mRNA in the lymphoblastoid cell lines (LCLs) of individuals from Family C after caffeine (7.5mM) treatment measured by semi-quantitative real-time RT-PCR.** Each sample has been normalised to the housekeeping gene, *GAPDH*, and calibrated to the lowest expressing untreated sample to show variation in transcript expression across individuals. Standard error bars represent standard error from the mean of four technical replicates for each PCR reaction. Click here for file

Additional file 7 **Stabilisation of*METRNL*mRNA in the lymphoblastoid cell lines (LCLs) of individuals from Family C after caffeine (7.5mM) treatment measured by semi-quantitative real-time RT-PCR.** Each sample has been normalised to the housekeeping gene, *GAPDH*, and calibrated to the lowest expressing untreated sample to show variation in transcript expression across individuals. Standard error bars represent standard error from the mean of four technical replicates for each PCR reaction. Click here for file

Additional file 8 **Stabilisation of*BMP6*mRNA in the lymphoblastoid cell lines (LCLs) of individuals from Family C after caffeine (7.5mM) treatment measured by semi-quantitative real-time RT-PCR.** Each sample has been normalised to the housekeeping gene, *GAPDH*, and calibrated to the lowest expressing untreated sample to show variation in transcript expression across individuals. Standard error bars represent standard error from the mean of four technical replicates for each PCR reaction. Click here for file
