# Supplementary material for: The application of nonsense-mediated mRNA decay inhibition to the identification of breast cancer susceptibility genes
Source: BMC Cancer. 2012 Jun 15;12:246. doi: 10.1186/1471-2407-12-246 (PMC3409022; doi:10.1186/1471-2407-12-246)
Supplement: Additional file 1 — Optimisation of caffeine concentration for lymphoblastoid cell lines (LCLs). Level of mRNA stabilisation for two biological replicates of SMAD4 in HT29 (A), BRCA1 in BRCA1 c.2681_2682delAA LCL (B), BRCA2 in BRCA2 c.6275_6276delTT LCL (C) and BRCA2 in BRCA2 c.539_541insAT LCL (D) after treatment with different concentrations of caffeine (untreated - 15mM). Error bars represent standard error of the mean. [file 1471-2407-12-246-S1.pdf]

Additional File 1: Primer sequences used for semi-quantitative real-time reverse transcriptase PCR of candidate genes identified with the GINI technique.

Gene	Forward or Reverse	5'-3' Sequence
GAPDH	Forward	CTGCACCACCAACTGCTTAG
GAPDH	Reverse	GTCTTCTGGGTGGCAGTGAT
PPARGC1A	Forward	GGCAGAAGGCAATTGAAGAG
PPARGC1A	Reverse	CATAGCTGTCTCCATCATCCC
WNT5A	Forward	AGGGTGATGCAGATAGGCAG
WNT5A	Reverse	GCGGTAGCCATAGTCGATGT
RAB3B	Forward	CAGAGCAGCTTGGGTTTGAT
RAB3B	Reverse	AGAGACGCGTGTTCTTGGAG
CD14	Forward	GGGATATAAGAGGCAGCCG
CD14	Reverse	CCTCTGAGCTCCGGACAG
METRNL	Forward	GTCTGCGCCGTGCC
METRNL	Reverse	AGGTGGATGGCTGAGTCCT
BMP6	Forward	GTGCAGACCTTGGTTCACCT
BMP6	Reverse	ACAAGCTCTTACAACCATATTCCTG
GRSF1	Forward	CTCCAGATAATAAGGGTGAAGCA
GRSF1	Reverse	GAAGCAATTAACCCAAAACACC
