# Supplementary material for: The application of nonsense-mediated mRNA decay inhibition to the identification of breast cancer susceptibility genes
Source: BMC Cancer. 2012 Jun 15;12:246. doi: 10.1186/1471-2407-12-246 (PMC3409022; doi:10.1186/1471-2407-12-246)
Supplement: Additional file 2 — Primer sequences used for semi-quantitative real-time reverse transcriptase PCR of candidate genes identified with the GINI technique. [file 1471-2407-12-246-S2.pdf]

A

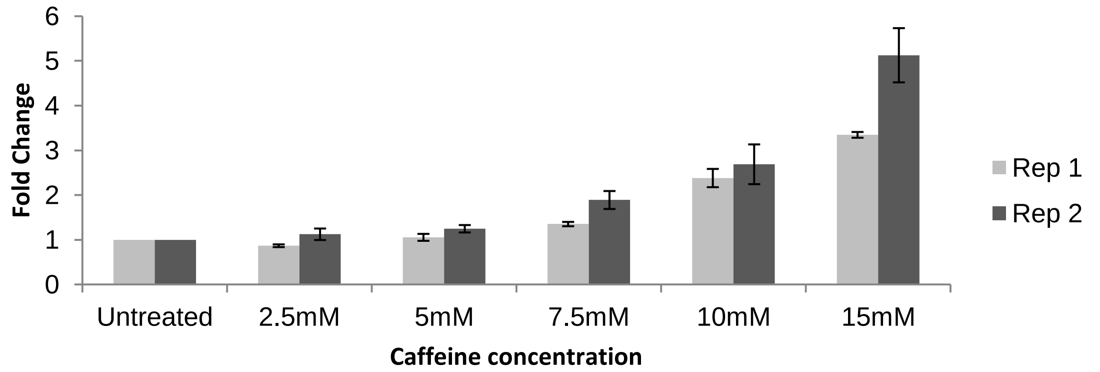

B

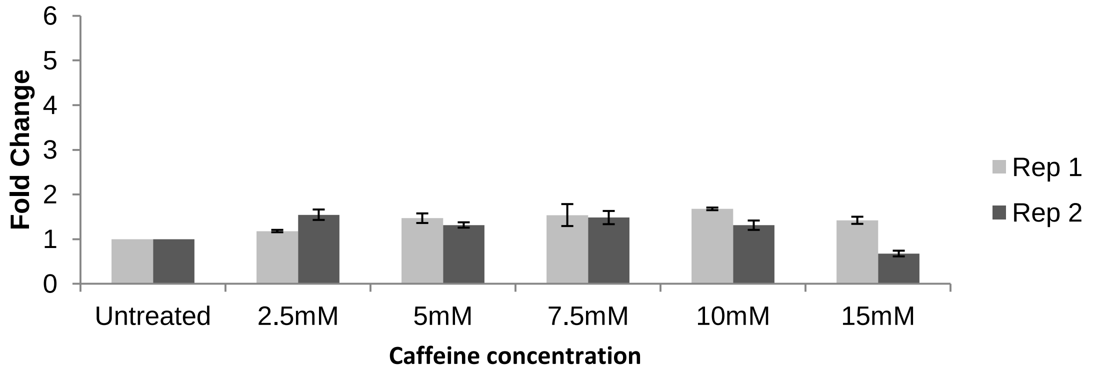

C

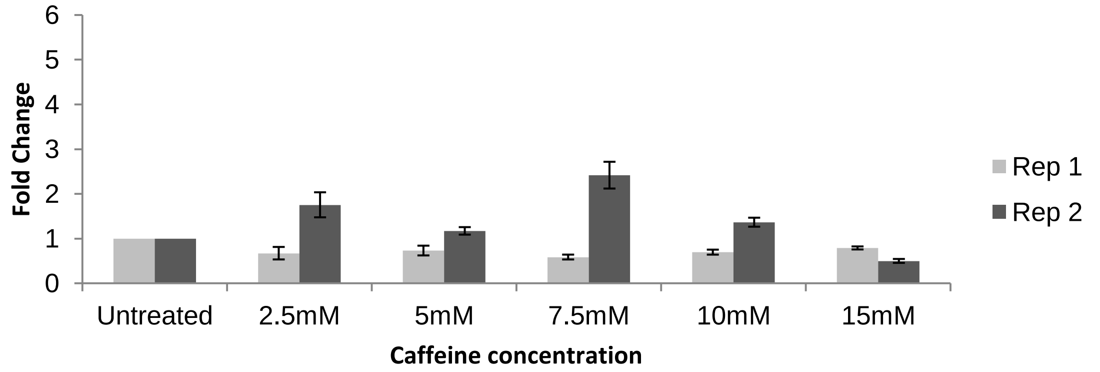

D

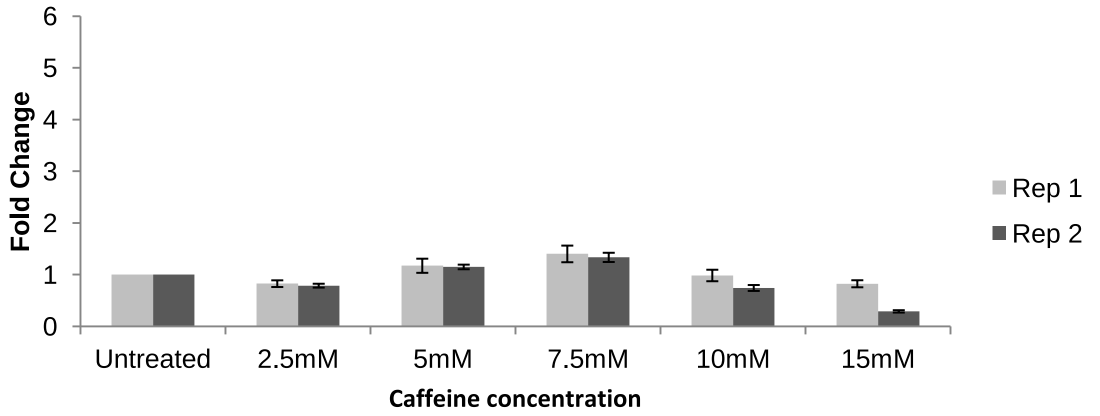

Additional File 2: Optimisation of caffeine concentration for lymphoblastoid cell lines (LCLs). Level of mRNA stabilisation for two biological replicates of *SMAD4* in HT29 (A), *BRCA1* in BRCA1 c.2681_2682delAA LCL (B), *BRCA2* in BRCA2 c.6275_6276delTT LCL (C) and *BRCA2* in BRCA2 c.539_541insAT LCL (D) after treatment with different concentrations of caffeine (untreated – 15mM). Error bars represent standard error of the mean.
