# Supplementary material for: The application of nonsense-mediated mRNA decay inhibition to the identification of breast cancer susceptibility genes
Source: BMC Cancer. 2012 Jun 15;12:246. doi: 10.1186/1471-2407-12-246 (PMC3409022; doi:10.1186/1471-2407-12-246)
Supplement: Additional file 6 — Stabilisation ofPPARGC1AmRNA in the lymphoblastoid cell lines (LCLs) of individuals from Family C after caffeine (7.5mM) treatment measured by semi-quantitative real-time RT-PCR. Each sample has been normalised to the housekeeping gene, GAPDH, and calibrated to the lowest expressing untreated sample to show variation in transcript expression across individuals. Standard error bars represent standard error from the mean of four technical replicates for each PCR reaction. [file 1471-2407-12-246-S6.pdf]

A

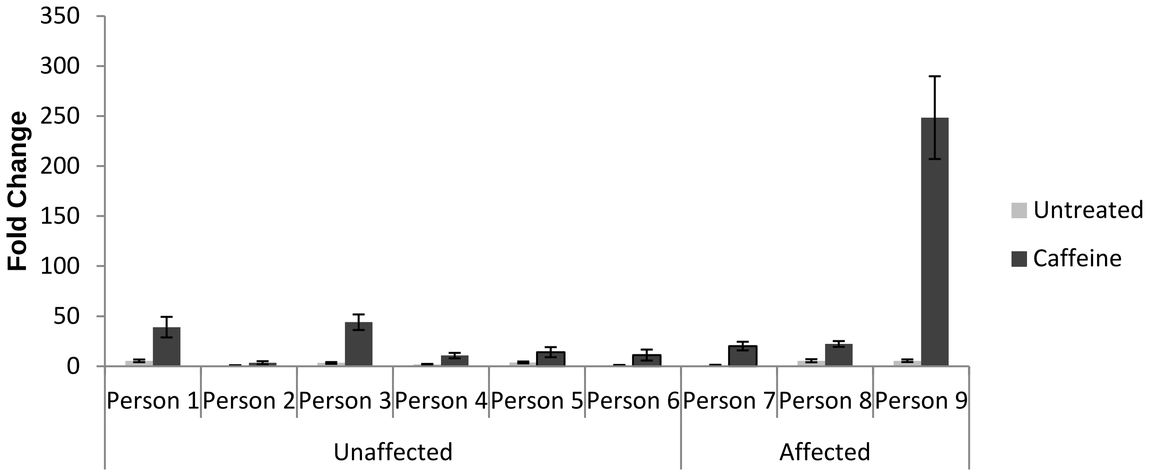

B

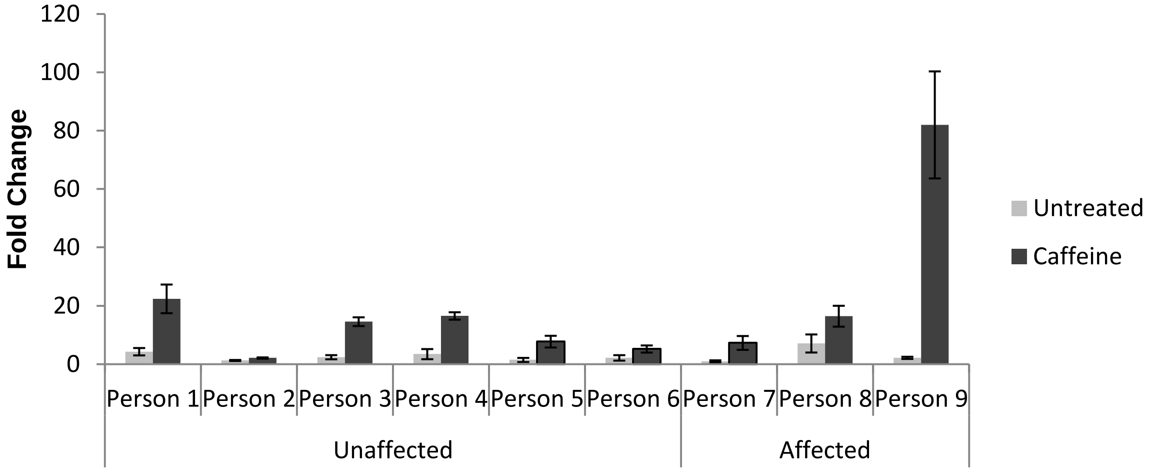

C

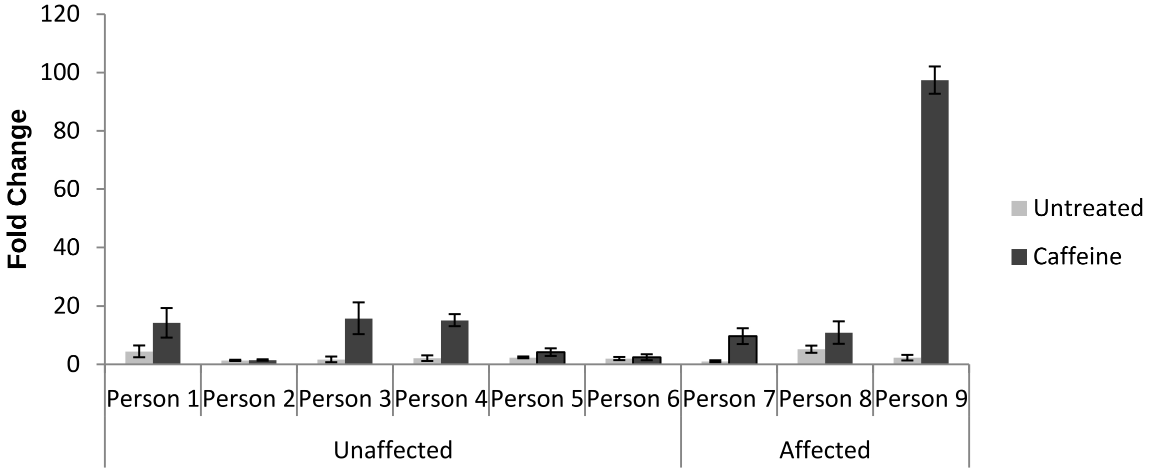

Additional File 6: Stabilisation of *PPARGC1A* mRNA in the lymphoblastoid cell lines (LCLs) of individuals from Family C after caffeine (7.5mM) treatment measured by semi-quantitative real-time RT-PCR. Each sample has been normalised to the housekeeping gene, *GAPDH*, and calibrated to the lowest expressing untreated sample to show variation in transcript expression across individuals. Standard error bars represent standard error from the mean of four technical replicates for each PCR reaction.
